# Data automated bag breathing unit for COVID-19 ventilator shortages

**DOI:** 10.1186/s40635-021-00419-2

**Published:** 2021-10-18

**Authors:** Aleksandra B. Gruslova, Nitesh Katta, Andrew G. Cabe, Scott F. Jenney, Jonathan W. Valvano, Tim B. Phillips, Austin B. McElroy, Robert K. LaSalle, Aydin Zahedivash, Van N. Truskett, Nishi Viswanathan, Marc D. Feldman, Richard B. Wettstein, Thomas E. Milner, Stephen Derdak

**Affiliations:** 1 Department of Medicine, UT Health San Antonio, 7703 Floyd Curl Drive, DTL 5.532U, San Antonio, TX 78229 USA; 2 Beckman Laser Institute, The University of California Irvine, Irvine, CA USA; 3 UT Austin Cockrell School of Engineering, The University of Texas, Austin, TX USA; 4 ThermoTek, Inc, Flower Mound, TX USA; 5 School of Health Professions, UT Health San Antonio, San Antonio, TX USA; 6 Dell Medical School, UT Austin, Austin, TX USA

**Keywords:** ABBU, Emergency resuscitator, Bag valve resuscitator, Lung injury, Acute respiratory distress syndrome, COVID-19, Ventilator shortage

## Abstract

**Background:**

The COVID-19 pandemic has caused a global mechanical ventilator shortage for treatment of severe acute respiratory failure. Development of novel breathing devices has been proposed as a low cost, rapid solution when full-featured ventilators are unavailable. Here we report the design, bench testing and preclinical results for an 'Automated Bag Breathing Unit' (ABBU). Output parameters were validated with mechanical test lungs followed by animal model testing.

**Results:**

The ABBU design uses a programmable motor-driven wheel assembled for adult resuscitation bag-valve compression. ABBU can control tidal volume (200–800 ml), respiratory rate (10–40 bpm), inspiratory time (0.5–1.5 s), assist pressure sensing (− 1 to − 20 cm H_2_O), manual PEEP valve (0–20 cm H_2_O). All set values are displayed on an LCD screen. Bench testing with lung simulators (Michigan 1600, SmartLung 2000) yielded consistent tidal volume delivery at compliances of 20, 40 and 70 (mL/cm H_2_O). The delivered fraction of inspired oxygen (FiO_2_) decreased with increasing minute ventilation (*V*_*E*_), from 98 to 47% when *V*_*E*_ was increased from 4 to 16 L/min using a fixed oxygen flow source of 5 L/min.

ABBU was tested in Berkshire pigs (*n* = 6, weight of 50.8 ± 2.6 kg) utilizing normal lung model and saline lavage induced lung injury. Arterial blood gases were measured following changes in tidal volume (200–800 ml), respiratory rate (10–40 bpm), and PEEP (5–20 cm H_2_O) at baseline and after lung lavage. Physiological levels of PaCO_2_ (≤ 40 mm Hg [5.3 kPa]) were achieved in all animals at baseline and following lavage injury. PaO_2_ increased in lavage injured lungs in response to incremental PEEP (5–20 cm H_2_O) (*p* < 0.01). At fixed low oxygen flow rates (5 L/min), delivered FiO_2_ decreased with increased V_E_.

**Conclusions:**

ABBU provides oxygenation and ventilation across a range of parameter settings that may potentially provide a low-cost solution to ventilator shortages. A clinical trial is necessary to establish safety and efficacy in adult patients with diverse etiologies of respiratory failure.

## Background

On January 31, 2020, the US Department of Health & Human Services announced a public health emergency related to a novel coronavirus, SARS-CoV-2, and the disease it causes, COVID-19 [1]. The early rapid spread of the COVID-19 pandemic resulted in a shortage of mechanical ventilators and accessory components (e.g., humidifiers, circuits, etc.) in many regions throughout the world [2–5]. In response to these shortages, a global surge in development and production occurred, including repurposing non-medical device assembly lines to manufacture quickly designed ventilators (e.g., FORD, GM, Virgin, etc.) [6–9].

As of March 2021, over 150 million COVID-19 cases have been identified leading to over 3.0 million deaths worldwide [10]. Among hospitalized patients, 30% require care at intensive care unit (ICU) and 29% or more of those require mechanical ventilation [11].

In response to the shortage of mechanical ventilators to treat COVID-19 patients, resuscitation bag-valve breathing devices were conceived as a potential solution for short-term emergency use. The FDA has classified these devices as "emergency resuscitators" to distinguish them from mechanical ventilators [12–16]. Our design uses a self-inflating resuscitation bag-valve, an automobile windshield motor, and lever arm to mimic manual hand bag-valve ventilation—along with essential operator controllable parameters: tidal volume (*V*_*T*_), respiratory rate (RR), inspiratory time (*T*_*I*_), positive end-expiratory pressure (PEEP) and patient-initiated breath pressure sensing. ABBU uses readily available components, low flow O_2_ sources, standard electrical power, and can be rapidly mass produced at lower cost ($2,000 estimated at 2021, ~ 5 h per unit production) compare to the full featured ICU ventilator ($25,000–$50,000).

The purpose of this study was to determine if ABBU can provide oxygenation and ventilation in a mechanical test lung and preclinical porcine model across a range of clinically relevant parameter settings.

## Methods

### Design: mechanical, electrical, software, safety

**Fig. 1:**
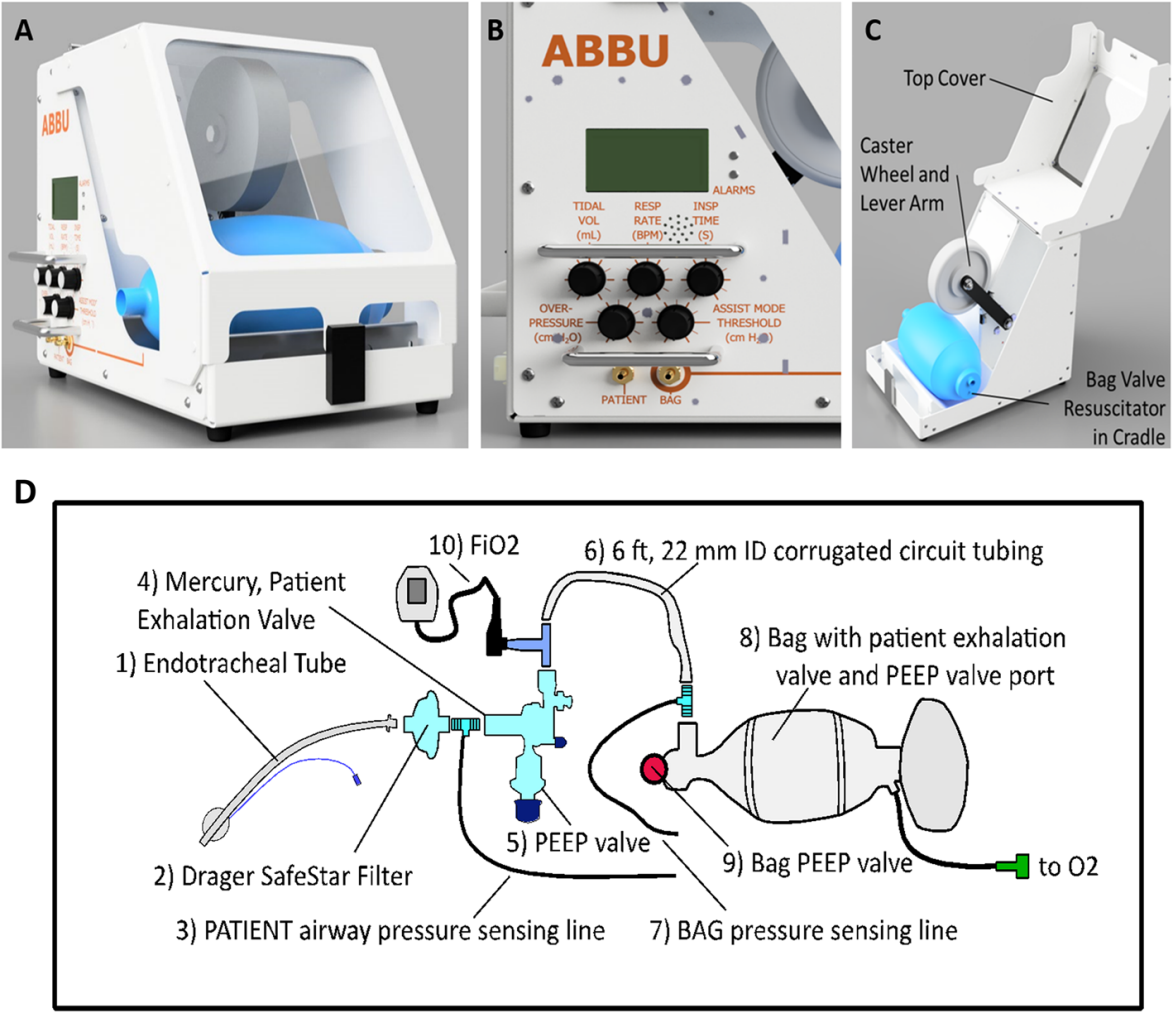
Automated Bag Breathing Unit (ABBU) and breathing circuit. **A** ABBU. **B** Front panel with LCD display and adjustment knobs. **C** ABBU cover openings. **D** ABBU breathing circuit used for testing. Porcine experiments added a sidestream ETCO_2_ analyzer between components 2 and 3)

ABBU was designed to replace manual ventilation of a bag valve resuscitator when a conventional ventilator device is unavailable (Fig. 1A, B). ABBU features include *V*_*T*_ (200–800 mL), RR (10–40 bpm), *T*_*I*_ (0.5–1.5 s), and adjustable patient-initiated breath sensing (− 1 to − 20 cm H_2_O). ABBU can use low flow oxygen (5–15 L/min) from widely available sources (e.g., concentrators, hospital wall-source, tanks, and liquid oxygen reservoirs).

ABBU senses the patient inspiratory effort below a software-calculated pressure threshold to trigger a breath. Patient–ABBU synchrony is facilitated by clinician titration of the triggering threshold. Auto-cycling can be reduced by increasing the triggering threshold (e.g., more negative). Conversely, ineffective triggering is reduced by decreasing the sensing threshold (e.g., less negative). Patients unable to trigger breaths (e.g., weakness, neuromuscular blocker-induced paralysis, central apneas) receive mandatory breaths at the set *V*_*T*_, RR, and *T*_*I*_. Total RR will be determined by the patient-triggered rate and the set rate.

ABBU provides visual and auditory alarms for circuit blockage, air-leaks, low pressure (e.g., disconnection), high airway pressure (50–70 cm H_2_O), motor, and electric failure. The audible power loss alarm has a backup battery. A high visibility enclosure facilitates rapid troubleshooting of the circuit and motor–bag interface (Fig. 1B). If ABBU fails, clinicians can quickly open the enclosure to access the bag and provide manual ventilation (Fig. 1C). This capability is a key safety feature of the ABBU design.

Figure 1D shows the breathing circuit components used in animal experiments. The patient exhalation valve (CPR-2 bag, Mercury Medical, Clearwater, FL) includes a manual adjustable PEEP valve. The resuscitator bag (adult Ambu® Spur® II bag, AMBU Inc., Columbia, MD) is centered in a cradle and secured on both ends by an elastic cord inside the unit. The bag PEEP valve (Ambu Disposable PEEP Valve, 0–20 cm H_2_O size) is set to 0 cm H_2_O, and PEEP is adjusted manually on a second PEEP valve interfaced to the patient exhalation valve. Two pressure sensing lines (3/16″ ID, 22 mm OD AirLife connector) are used for circuit pressure monitoring and breath triggering assist. An FiO_2_ analyzer (MaxO^2^ + AE, Maxtec, Salt Lake City, UT) was interfaced into the breathing circuit for all animal experiments.

### Bench testing

A Michigan adult dual-lung simulator (Model 1600, Michigan Instruments, USA) and Ventilator Validation System (VVK100-SYS, BIOPAC Systems, Goleta, CA) was used to validate ventilator parameters. ABBU was tested at compliances of 20, 40 and 70 mL/cm H_2_O with resistances 5–50 cm H_2_O/(L/s). V_T_ of 200, 400, 600 and 800 mL were tested across a range of compliances and resistance levels. For performance experiments, RR was set at 15 bpm and the PEEP was set at 15 cm H_2_O. Twenty breath cycles were collected for each measurement and processed to validate measures of V_T_, pressure, T_I_, RR, and confirm PEEP value.

Durability and V_T_ stability of different brand resuscitation bags: AMBU (SPUR II, Ambu, Columbia, MD), HUDSON (RCI 5387, Teleflex, Morrisville, NC), MEDLINE (CPRM1116, Medline, Northfield, IL), Mercury (CPR-2, Mercury Medical, Clearwater, Florida) were evaluated on mechanical test lungs (SmartLung 2000, IMT Analytics, Buchs, Switzerland) at maximum RR (50 bpm) and *T*_*I*_ of 0.5 s continuously over 7 days. Cardone electric motors (Model 85–3024, Cardone Industries, Ontario, CA) were operated continuously for > 30 days to assess durability.

### Animal testing

Animal procedures were approved by the Institutional Animal Care and Use Committee at the University of Texas Health Science Center at San Antonio. Studies were performed on 6 healthy female pigs (Berkshire, 50.8 ± 2.6 kg). Pigs were sedated via tiletamine–zolazepam (Telazol,4–8 mg/kg IM), Zylazine (1–2.2 mg/kg IM), and 3–4% Isoflurane, followed by endotracheal intubation and maintenance on 0.5–3% Isoflurane. Body temperature was kept in the normal range (38–39 °C) by heated pad. Arterial pulse pressure was monitored by a micromanometer pressure sensor in the descending thoracic aorta. After collection of baseline blood samples, ABBU was connected to the proximal end of the endotracheal tube by a 90-degree adapter plugged into the breathing circuit, which included the FiO_2_ analyzer and side-stream ETCO_2_ analyzer (Fig. 1D).

ABBU settings were changed in accordance with the experimental protocol. T_I_ was kept constant (1 s) during the entire experiment. Baseline testing was performed on healthy lungs, followed by testing on saline injured lungs. Neuromuscular paralysis was used as needed (vecuronium, IV, 0.1–0.2 mg/kg). Heart rate (HR), blood pressure (BP) and body temperature (rectal) were monitored continuously. Pigs were euthanized using Euthasol solution (pentobarbital sodium and phenytoin sodium, IV, 100 mg/kg) following completion of experiments (6–8 h).

Saline lung lavage was performed as previously described [17]. In brief, warmed saline (30 mL/kg) was poured into the lungs through a funnel. As arterial pressure fell below 50 mm Hg, lavage fluid was drained passively. The animal was reconnected to ABBU with an O_2_ flow rate of 15 L/m and RR was adjusted to maintain arterial pH > 7.25. Lavages were repeated until partial pressure of oxygen (PaO_2_) was < 100 mm Hg [13.3 kPa] for 30 min.

Arterial blood samples were analyzed by CG4 + cartridges (iSTAT analyzer, Abbott, IL, USA). Blood gas responses for different V_T_, RR and PEEP were compared with their respective baselines for normal and lung injury model. Parameters: FiO_2_ (%), HR (bpm), RR (bpm), ETCO_2_ (mm Hg), SpO_2_ (%), and BP (mm Hg), were recorded concurrent with blood sample collection.

### Statistical analysis

Data in graphs is shown as mean ± SE. Two-tail *T* test and one-way ANOVA were used for all comparisons. A value of *p* < 0.05 was considered statistically significant. Pearson Correlation Coefficient (PCC) was computed to test correlation between two variables.

## Results

### Bench testing

#### Durability testing of bags and motors

**Fig. 2:**
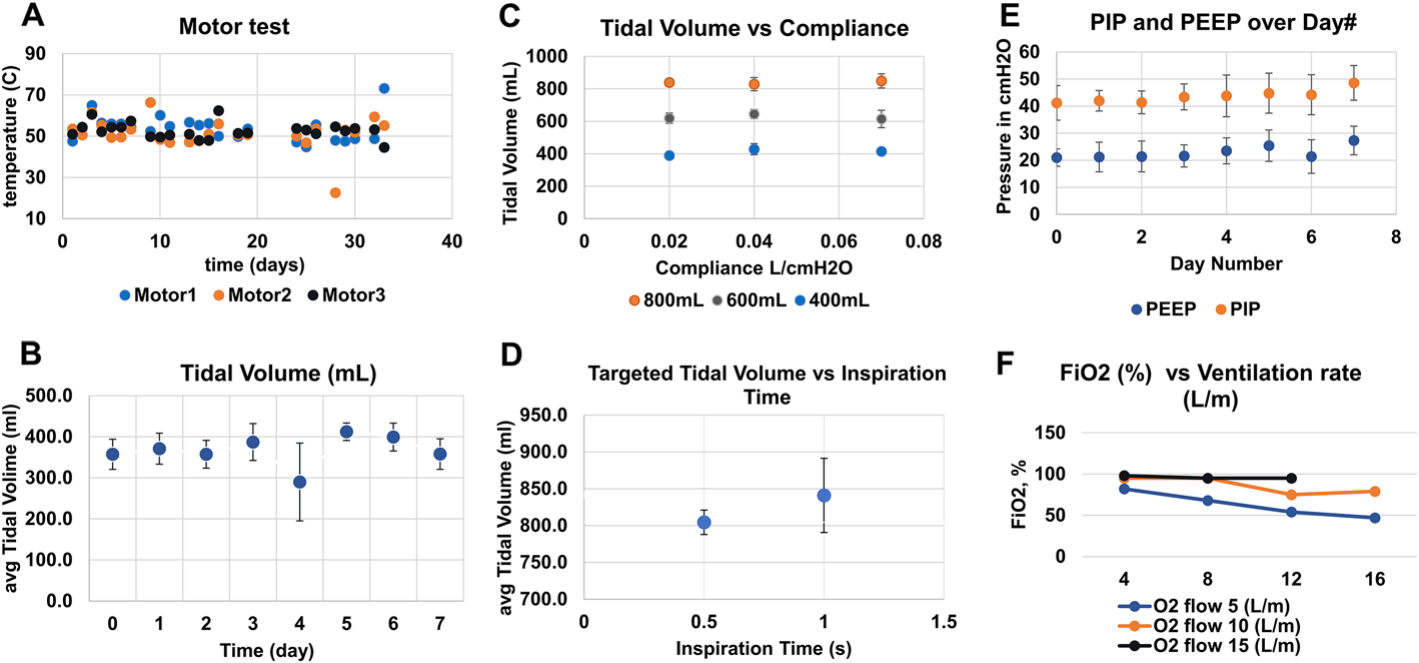
Bench testing results with the lung simulator. **A** Cardone motors test at V_T_ of 800 mL and RR of 50 bpm for 32 days. **B** Tidal volume consistency of the AMBU Spur II bag (n = 7) operating continuously for 7 days. **C** Tidal volume delivery measured at varying compliances (0.02, 0.04, 0.07 L/cm H_2_O) for 3 tidal volumes of 200, 400 and 800 mL, and 1 s for T_I_. **D** Tidal volume performance as a function of changing inspiratory time at a fixed compliance of 0.02 L/cm H_2_O at a delivered tidal volume of 800 mL. **E** PEEP and PIP values during 7 days of continuous operation (*V*_*T*_—800 mL, respiratory rate—50 bpm. **F** Changes in FiO_2_ with increase in minutes ventilation rate (*V*_*T*_—400 mL)

Three Cardone motors were continuously operated at constant *V*_*T*_ (800 mL) and RR (50 bpm) for 32 days. The motor temperature was monitored. The testing was discontinued after Motor 1 overheated (up to 73 °C) and stopped operating (Fig. 2A). Only one of the 5 tested motors reached this temperature.

In addition, the performance of four brands of bags (AMBU Spur II, Hudson, Medline, Mercury) was tested with ABBU running continuously for 7 days at V_T_ 800 ml and RR 50 bpm. Data for each bag was collected at V_T_ of 400 mL. The correlation slope and standard deviation were calculated to indicate when bag performance started to decline. A slope correlation closest to 1 indicates bag is able to achieve the targeted V_T_ for all parameters in the test matrix. The AMBU Spur II bag performed the best (correlation slope between 0.93 and 1.03, data not provided). Figure 2B shows experiments with seven Spur II AMBU bags. Values computed from the Ventilator Validation System (BIOPAC System, Inc.) data were consistent with the set controls on the ABBU instrument. The data demonstrates the consistent performance of AMBU Spur II bag (400 mL *V*_*T*_, 15 bpm RP, 1 s *T*_*I*_) over 7 days of continuous ABBU operation. At day 4, there was a significant variation in V_T_ resulting from a shift of bag position in the cradle. AMBU bag degradation (scratches, loss of elasticity, loss of compliance) was observed after continuous operation (RR 50 bpm, *T*_*I*_− 0.5 s, *V*_*T*_ 800 mL) starting at day 4; however, performance was still adequate. Performance declined after 7 days, after which replacing the bag is recommended.

#### Accuracy of controls of instruments

Targeted V_T_ of 400 mL was consistently delivered (SD ≤ 50 mL) to the Michigan test lung at varying compliances (20, 40, 70 mL/cm H_2_O) and T_I_ settings (0.5, 1 s) (Fig. 2C, D).

PEEP and PIP showed minimal variation during continuous operation of ABBU for 7 days at *V*_*T*_ of 800 mL and RR of 50 bpm (Fig. 2E).

FiO_2_ decreased significantly with increasing RR at a constant *V*_*T*_ (400 mL) and fixed oxygen flow rate of 5 L/m (Fig. 2F).

### Animal testing

ABBU was tested in a porcine model at baseline and following saline lavage lung injury. Data was obtained sequentially on the same animal over 6–8 h. HR, BP and body temperature were maintained within physiological levels throughout experimentation.

#### Normal lung

**Fig. 3:**
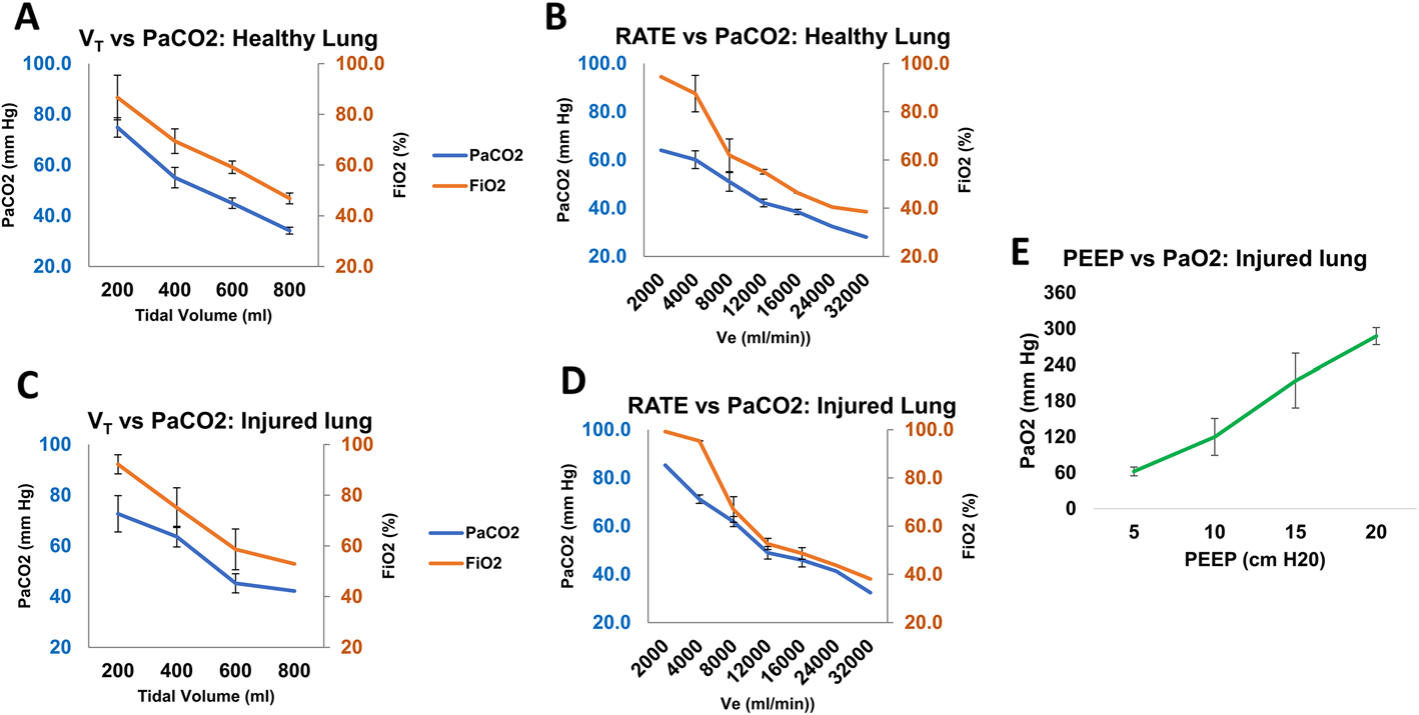
Porcine study results (*n* = 6). **A**, **B** Variation of PaCO_2_ and PaO_2_ by adjusting Tidal volume, Respiratory Rate or PEEP in normal or **C**–**E** injured lung porcine model. T_I_ (1 s) and oxygen flow rate (5 L/min) were kept constant during experiments. All results are mean ± SE

Before experiments, the blood gas and hemodynamic responses from switching to ABBU were compared with the veterinary ventilator baseline (Narkomed 2B, Drager, Germany). Switching from the veterinary ventilator (FiO_2_ 100%) to ABBU (FiO_2_ 73%) caused a decrease in PaO_2_ from 467.5 ± 25.8 mm Hg [62.3 ± 3.4 kPa] to 307.3 ± 51.9 mm Hg [41 ± 6.9 kPa] attributed to the difference in FiO_2_ between the two devices (Table 1). The blood gas responses at different *V*_*T*_ and RR were compared with their respective baseline values (Fig. 3) at constant *T*_*I*_ (1 s), PEEP (5 cm H_2_O), and flow rate (5 L/min). Mean PaCO_2_ in arterial blood at baseline *V*_*T*_ (200 mL) was 74.8 ± 3.8 mm Hg [10 ± 0.5 kPa]. Figure 3A demonstrates that increasing *V*_*T*_ and with fixed RR at 20 bpm, lowered PaCO_2_ to physiological level (≤ 40 mm Hg [5.3 kPa]) in all animals (*p* < 0.001). Changes from high to low PaCO_2_ (e.g., low to high *V*_*E*_) was associated with significant decreases in FiO_2_ (0.998 PCC) similar to test lung data using a fixed oxygen flow rate. Increases in *V*_*E*_ decreased delivered FiO_2_ at all combinations of *V*_*T*_ and RR. The response of PaCO_2_ to changes in RR, while keeping V_T_ constant is shown in Fig. 3B.

**Table 1:** Gas exchange and hemodynamics

Ventilator	FiO_2_, %	CO_2_, mm Hg [kPa]	SpO_2_, %	pH	PaCO_2_, mm Hg [kPa]	PaO_2_, mm Hg [kPa]	SaO_2_, %
Veterinary ventilator	100 ± 0	41.5 ± 0.8 [5.5 ± 0.1]	97.8 ± 0.8 [13.0 ± 0.1]	7.5 ± 0.01	45.5 ± 1.9 [6.1 ± 0.3]	467.5 ± 25.8 [62.3 ± 3.4]	100 ± 0
ABBU	72.7 ± 10.1^a^	43.8 ± 2.6^b^ [5.8 ± 0.3]	95.8 ± 0.9^b^ [12.8 ± 0.1]	7.4 ± 0.1^b^	51.3 ± 8.2^b^ [6.8 ± 1.1]	307.3 ± 51.9^a^ [41.0 ± 6.9]	100 ± 0

Summary of O_2_ and CO_2_ exchange with the veterinary ventilator and ABBU. Two-tail *T* test was used for comparisons. All results are mean ± SE

^a^*p* < 0.05 vs veterinary ventilator; ^b^*p* > 0.05 vs veterinary ventilator

#### Saline lavage lung injury

Hypoxemia following saline lavage was confirmed in all six pigs. The average arterial PaO_2_, PaCO_2_ and pH at baseline was 69.5 ± 8.6 mm Hg [9.3 ± 1.1 kPa], 43.1 ± 2.2 mm Hg [57.5 ± 0.3 kPa] and 7.4 ± 0.02, respectively.

Similar to pre-lavage baseline, an increase in V_T_ while maintaining a constant RR (20 bpm) led to a significant decrease in arterial PaCO_2_ level (*p* < 0.005) and FiO_2_. Mean PaCO_2_ (mm Hg [kPa]) at 200 mL, 400 mL and 600 mL was 72.7 ± 7.1 [9.7 ± 0.9], 63.7 ± 4.1 [8.5 ± 0.5] and 45.3 ± 3.8 [6.0 ± 0.5], respectively. Increasing RR, demonstrated a similar decline in PaCO_2_ (*p* < 0.001) and decrease in FiO_2_ (0.984 PCC).

In 5 out 6 pigs, increasing PEEP effectively improved oxygenation of the saline injured lung while maintaining a constant RR (20 bpm), *V*_*T*_ (400 mL) and flow rate (5 L/min) (Fig. 3E). Incremental PEEP steps from 5 to 20 cm H_2_O led to significant increase in PaO_2_ from 62.3 ± 7.4 mm Hg [8.3 ± 0.99 kPa] to 287.5 ± 14.2 mm Hg [38.3 ± 1.9 kPa] (*p* < 0.01).

#### ABBU synchronous operation testing

**Fig. 4:**
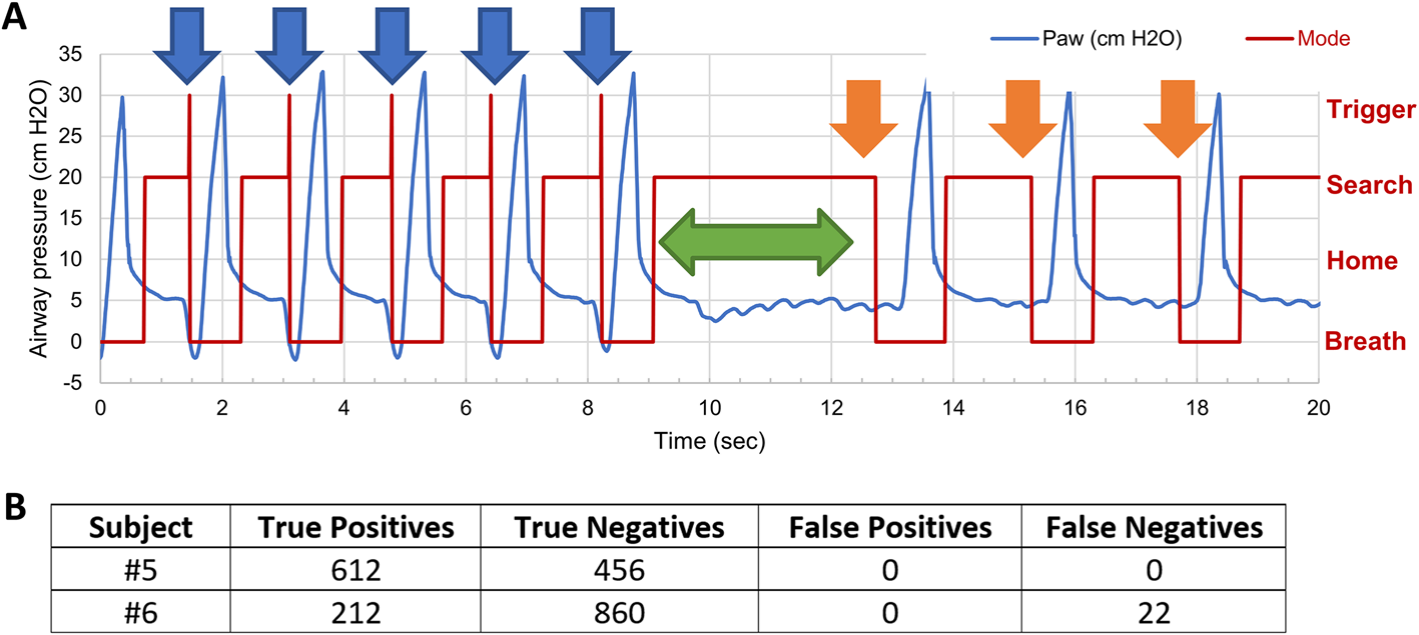
ABBU breath triggering data. **A** Representative pressure–time scalar (blue) during one porcine experiment. The first five breaths are trigger assisted (blue arrows), followed by a two respiratory cycle pause (green arrow), followed by three control breaths (orange arrows). Mode = Breath means a mechanical breath is being is delivered, Mode = Home means the motor is idle and Paw is below PEEP, Mode = Search is when it calculates PEEP and is searching for a patient effort, and Mode = Trigger means a breath assist is triggered. **B** Summary of trigger assist algorithm (*n* = 2). Note that Subject #6 had 22 false negatives (ineffective triggering) attributed to inability of the algorithm to calculate the sensing threshold below PEEP during periods of rapid breathing (50–60 bpm)

A breath-triggering software algorithm was developed during the first four pig studies, with over 1000 breaths analyzed with each experiment. After each study, the algorithm was tuned. A “true positive**”** is defined as the patient initiating a breath (e.g., triggered-assisted breath) and ABBU delivering an assist within 160 ms. A “true negative” (e.g., control breath) is defined as the patient not initiating a breath and ABBU delivering a breath according to the rate setting. A “false positive” is defined as ABBU delivering a breath at a time the patient did not initiate (e.g., false triggering or auto-cycling). A “false negative**”** (e.g., ineffective triggering) is defined as the patient initiating a breath and ABBU not delivering an assist. False positives occurred during suction and airway disconnect. To remove these false positives, we added two blanking times, when the algorithm does not look for patient assist. The first blanking time is shown in Fig. 4A as the time between mode = Breath (start of delivered breath) and mode = Home (end of delivered breath when motor returns to home) eliminates the period, while the bag is being compressed and the airway pressure is changing rapidly. Mode = Home is when the motor is home, the airway pressure is below PEEP. Mode = Search is when the motor is home, the airway pressure is above the previous PEEP, the algorithm calculates PEEP for this breath, and it searches for a patient effort. The second blanking time between Mode = Home and Mode = Search eliminates the false positives that would occur on suction and airway disconnect. Mode = Trigger in Fig. 4A signifies the algorithm triggered a synchronous breath, because the airway dropped below the PEEP for this breath. In Fig. 4A, an asynchronous breath occurs when the algorithm cycles through modes Breath–Home–Search (orange arrows), and a synchronous breath cycles through modes Breath–Home–Search–Trigger (blue arrows). The two blanking periods also eliminated run-away, where ABBU delivered breaths above 60 bpm. However, the blanking periods causes false negatives if the patient attempts to breathe faster than 60 bpm. During the first four pig studies, false negatives occurred when the patient attempted to breathe at a rate close to the asynchronous rate set on ABBU. To eliminate these false triggers, the algorithm was modified to include a two respiratory cycle pause after a triggered breath before delivering a control breath (see the time from 9 to 13 s in Fig. 4A). A rapid increase in patient PEEP valve of more than 5 cm H20 per breath causes false positives. Similarly, a rapid decrease in patient PEEP valve causes false negatives. These observations led to the recommendation to adjust the patient PEEP valve slowly, so the algorithm operates properly during the change.

The final version of the ABBU triggering algorithm (e.g., assist-control mode) was tested in 2 pigs (Fig. 4). The sensing threshold was set at -5 cm H_2_O, and data was collected as the animal went from light anesthesia (spontaneous breathing) to deep anesthesia (paralysis). Tidal volume was adjusted from 200 to 800 mL. PEEP valve was set at 5 cm H_2_O. The algorithm effectively calculated PEEP for pressure threshold sensing unless there was applied suction, circuit leaks, or rapid respiratory rates exceeding 50 bpm (Fig. 4, Panel B, Subject #6).

## Discussion

The shortage of mechanical ventilators due to the COVID-19 pandemic has led to attempts to repurpose hand-operated AMBU bags into automated bag-compression devices [6–8, 11–16]. In 2020, our group developed and tested the Automated Bag Breathing Unit (ABBU), to assist with the shortage of conventional ventilators [1, 2]. ABBU uses widely available resuscitation bags and circuit components and can be quickly mass-produced to potentially mitigate conventional ventilator shortages. An ABBU training manual and instructional video were tested by respiratory therapy students at the University of Texas Health Sciences Center at San Antonio. A post training survey indicated that students could quickly perform circuit and basic operation set up.

ABBU is not a full-featured ICU ventilator, but a device that provides automated compression of a bag valve resuscitator. The FDA classifies these devices as "emergency resuscitators" and they typically provide controlled ventilation with a fixed oxygen flow rate, adjustable RR and V_T_, manual PEEP valve, and basic alarms, such as high airway pressure or power failure [19, 20].

To our knowledge, ABBU is the only resuscitator providing a software-based pressure-sensing algorithm with adjustable triggering thresholds. This is an important feature of ventilation in patients with acute respiratory failure or when weaning patients from ventilation [21]. Despite a growing number of approved FDA Emergency Use Authorization (EUA) resuscitators, few have published specifications or pre-clinical testing results, and none have reported clinical trials in patients [22–26]. Here we report ABBU is capable of providing physiological gas exchange in a short-term (6–8 h) adult-size porcine model of normal and saline lavage lung injury. A saline lavage injury model was chosen for simplicity and reproducibility. Saline lavage causes surfactant washout with readily recruitable lung and rapid recovery but does not reflect the severity or heterogeneity of clinical acute respiratory distress syndrome [17, 26]. As expected, PaO_2_ increased with incremental PEEP and PaCO_2_ decreased with incremental minute ventilation by adjusting V_T_ or RR.

It is important to understand that automated resuscitators, including ABBU, have significant limitations compared to fully functional ICU ventilators [9, 20, 27, 28]. Due to the use of a fixed low flow oxygen source, delivered FiO_2_ *decreases* with increases in V_T_ or RR and may be a significant factor contributing to oxygen desaturation in patients. ABBU has no capacity for automated flow augmentation or leak compensation, such that PEEP decays during the exhalation phase. PEEP decay may be clinically significant in patients with long exhalation times, bronchopleural fistulas, or endotracheal cuff-leaks, resulting in loss of lung recruitment.

Additional limitations of ABBU and similar emergency resuscitators include a lack of measuring actual V_T_ delivery (e.g., set bag V_T_ plus spontaneous breath V_T_) which may be significantly greater than the clinician set *V*_*T*_ (or less in the setting of air leaks). There is no automated inspiratory or expiratory pause feature to assess inspiratory plateau pressure or auto PEEP, respectively. In contrast to ICU ventilators, there are no pressure, volume, or flow graphics to assess respiratory mechanics or patient–ventilator synchrony. Patient work of breathing and ABBU–patient synchrony assessment could not be readily reproduced in this anesthetized animal model and should be evaluated in clinical trials.

The ABBU design currently does not have an integrated battery backup for use as a transport device. However, in case of electrical or motor failure, the AMBU bag may be removed from the enclosure and used manually. This feature is an advantage over resuscitation devices that rely on a continuous source of compressed air. Durability of the ABBU device may be limited by the lifespan of the electric motor and AMBU bag (approximately 30 and 7 days of continuous operation, respectively).

ABBU's limitations are inherent to the simplicity and low-cost design goal of achieving rapid mass production in a ventilator shortage scenario. These deficiencies are potentially addressable by close patient monitoring to include use of pulse oximetry, end-tidal CO_2_, FiO_2_ analyzer, and a V_T_ respirometer. At the time of this writing, an application for FDA Emergency Use Authorization has been submitted and is pending review.

## Conclusions

The ABBU emergency resuscitator supports short term oxygenation and ventilation in an animal model across a range of parameter settings that may potentially provide a low-cost solution to adult ventilator shortages. Clinical trials of ABBU (and similar emergency resuscitation bag devices) are necessary to establish safety and efficacy before use in patients with diverse etiologies of respiratory failure.

## Data Availability

The data sets generated and analyzed during the current study are available from the corresponding author on reasonable request.

## Abbreviations

ABBU: Automated Bag Breathing Unit
BP: Blood pressure
ETCO_2_: End-tidal carbon dioxide
FiO_2_: Inspired oxygen
HR: Heart rate
ICU: Intensive care unit
PaCO_2_: Partial pressure of carbon dioxide
PaO_2_: Partial pressure of oxygen
PCC: Pearson correlation coefficient
PEEP: Positive end-expiratory pressure
RR: Respiratory rate
T_I_: Inspiratory time
V_E_: Minute ventilation
V_T_: Tidal volume
